# Successful endovascular treatment of post-traumatic subclavian arteriovenous fistula

**DOI:** 10.1093/jscr/rjae764

**Published:** 2024-12-02

**Authors:** Veysel Embel, Emmanuel Ihionkhan, Charles Lu, Vincent Marcucci, Vikalp Jain

**Affiliations:** Department of Surgery, Hackensack Meridian Health Jersey Shore University Medical Center, 1945 Route 33, Neptune, NJ 07756, United States; Department of Surgery, Hackensack Meridian Health Jersey Shore University Medical Center, 1945 Route 33, Neptune, NJ 07756, United States; Department of Surgery, Hackensack Meridian Health Jersey Shore University Medical Center, 1945 Route 33, Neptune, NJ 07756, United States; Department of Surgery, Hackensack Meridian Health Jersey Shore University Medical Center, 1945 Route 33, Neptune, NJ 07756, United States; Department of Surgery, Hackensack Meridian Health Jersey Shore University Medical Center, 1945 Route 33, Neptune, NJ 07756, United States

**Keywords:** arteriovenous fistula, endovascular repair, subclavian artery, traumatic fistula

## Abstract

Subclavian arteriovenous fistulas (AVFs) are rare entities, mostly reported as a result of traumatic and iatrogenic injuries. In the literature, congenital subclavian AVFs are also presented. Diagnosis of traumatic AVF may present challenges given the variable clinical presentation, varying location, and difficulty to locate on imaging. If left untreated, it can lead to high-output heart failure. This underlies the importance of understanding the nature of the disease, timely diagnosis, and treatment in order to prevent increased morbidity and mortality. We report a case of a patient having a traumatic left subclavian AVF formation after clavicle and rib surgery, which was successfully treated with a covered stent.

## Introduction

Arteriovenous fistulas (AVFs) are anomalous communications between arteries and veins. These may be congenital or acquired, with the latter as a sequela of penetrating or blunt trauma or iatrogenic injuries associated with endovascular or surgical procedures [1, 2]. Fistulas that are left untreated in the setting of a traumatic event can ultimately lead to complications that include high output heart failure, pain, swelling, or skin breakdown. The incidence of traumatic AVFs and pseudoaneurysms are described in the literature as approximately 6.8%–21.2% more commonly located in the lower extremities near long bones [3]. Here, we describe a case in which an initial steroid injection for rotator cuff injury led to infection and subsequent surgical procedures resulted in the development of a fistulous connection between the subclavian artery and subclavian vein.

## Case presentation

**Figure 1:**
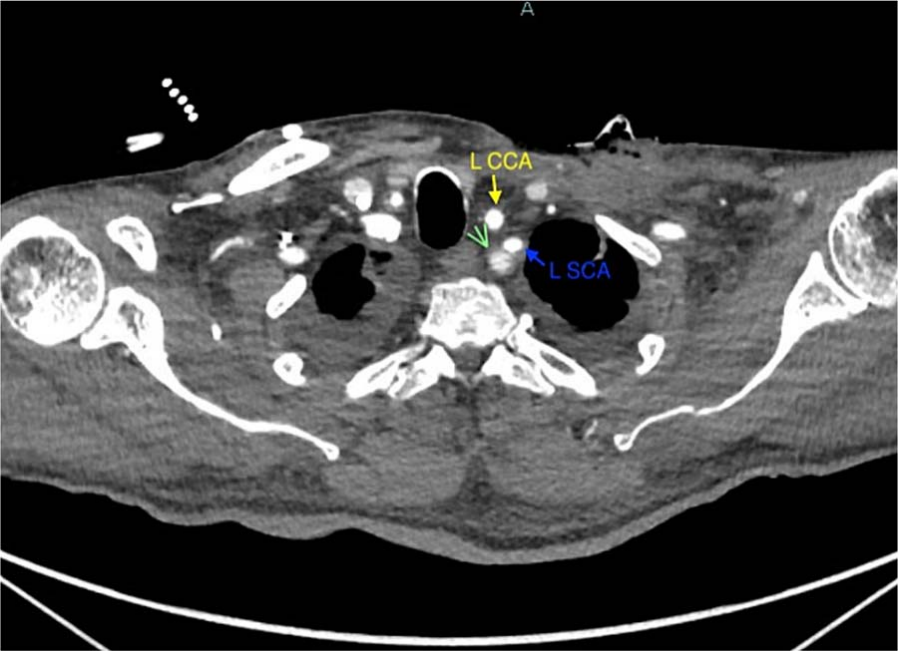
Demonstration of pseudoaneurysm of the proximal left subclavian artery on axial image (green arrow: L SCA PSA, blue arrow: left subclavian artery, yellow arrow: left common carotid artery).

**Figure 2:**
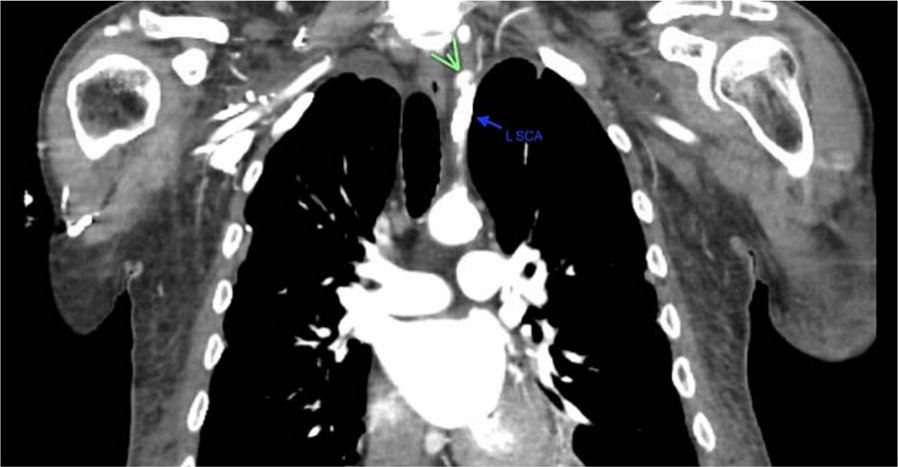
Demonstration of pseudoaneurysm of the proximal left subclavian artery on axial image (green arrow: L SCA PSA, blue arrow: left subclavian artery).

**Figure 3:**
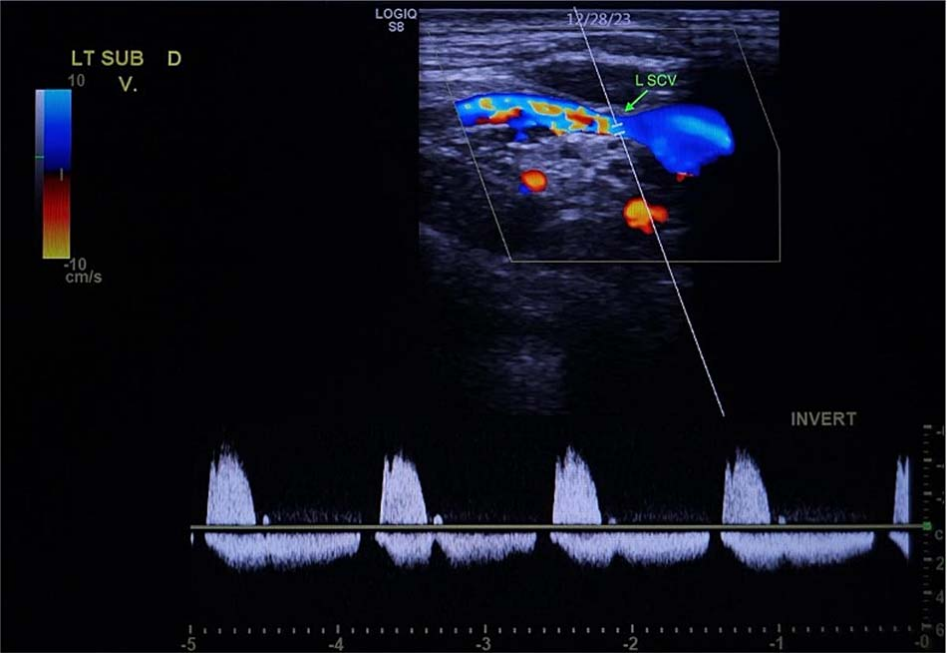
Demonstration of arterial waveform in the left subclavian vein on Duplex ultrasound (green arrow: left subclavian vein).

**Figure 4:**
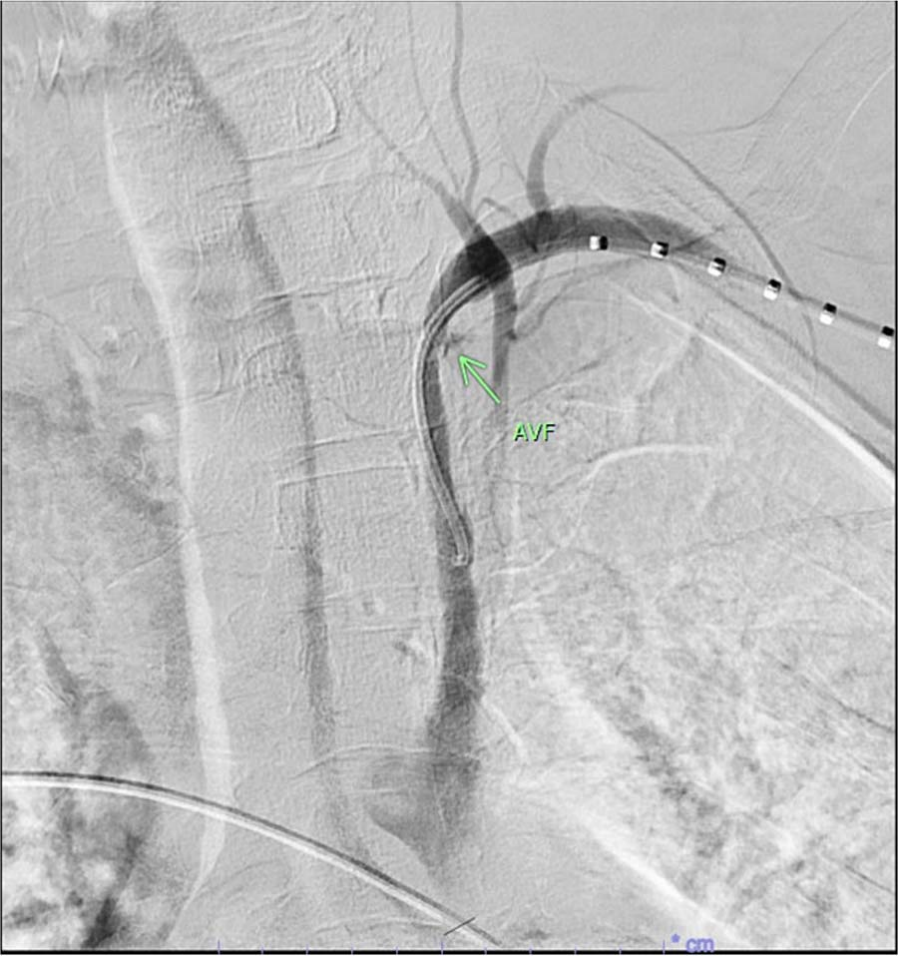
Demonstration of fistulous connection between subclavian artery and vein (green arrow).

**Figure 5:**
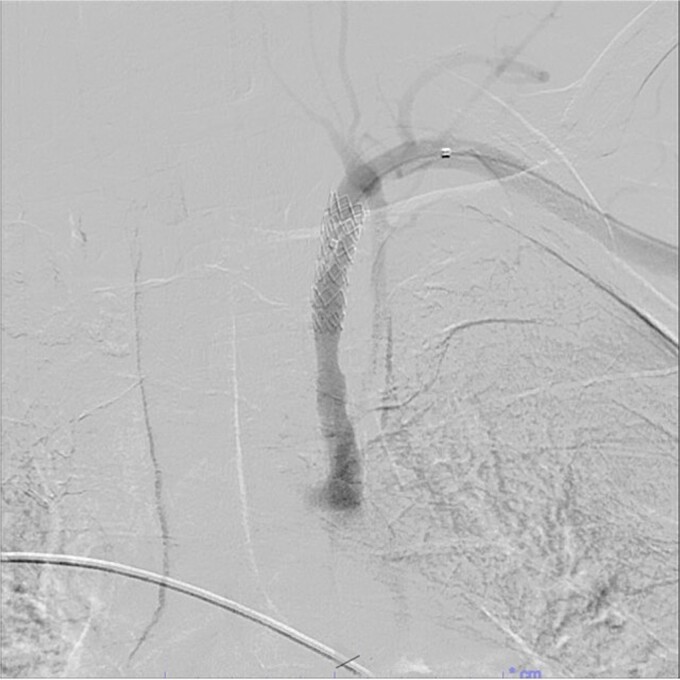
Demonstration of covered stent placement and resolution of the fistulous connection.

A 67-year-old male with a medical history significant for hypertension, diabetes, coronary artery disease, carotid artery stenosis, osteoarthritis, and 50 pack-year smoking history presented initially to an orthopedic surgery office for evaluation of left shoulder pain after exercising. The patient was found to have an underlying rotator cuff injury and was subsequently treated with cortisone injection. The patient subsequently developed septic arthritis, ultimately requiring incision and drainage of the abscess and resection of the sternoclavicular joint, partial first rib, and partial clavicle. The patient required multiple washouts and debridement procedures, ultimately leading to septic shock and bacteremia requiring close observation in the surgical intensive care unit. A computed tomography angiogram (CTA) of chest was performed at the time and revealed a subcentimeter penetrating ulcer, arising from the proximal left subclavian artery likely secondary to surgical trauma, which was observed nonoperatively given the patient critical status in the intensive care unit (ICU) (Figs 1 and 2). During the ICU course, the patient developed left upper extremity swelling A left upper extremity duplex ultrasound subsequently revealed a nonocclusive deep vein thrombosis of the subclavian vein and also showed resolution of a pseudoaneurysm (PSA) of the subclavian artery. The patient was started on anticoagulation at this time for a deep vein thrombosis (DVT). The patient was ultimately discharged upon resolution of his acute infection to a rehabilitation facility. During the third-month follow-up office visit, the fistulous connection between the subclavian artery and vein was found incidentally on left upper extremity duplex ultrasound (Fig. 3). Physical exam of the patient was otherwise unremarkable with palpable upper extremity pulses and the patient did not appear to have any symptoms related to the fistula including upper extremity swelling or open wounds. The patient was scheduled for an elective repair of the fistula via endovascular stent graft placement. The patient was brought to the operating room, and the radial artery was accessed with a micropuncture device. Radial artery access was our choice to intervene given the location of fistula. When left upper extremity angiography was performed, it revealed a blush of contrast from proximal subclavian artery, revealing a small fistula between subclavian artery and subclavian vein (Fig. 4). Subsequently, a 6 × 29 mm balloon-mounted stent graft was used to cover the fistula. Completion angiography was performed showing adequate seal with resolution of the fistula (Fig. 5). The patient tolerated the procedure well and was subsequently discharged from the hospital the same day. He was started on antiplatelet therapy post-operatively and continued oral anticoagulation for the DVT. The patient was seen in the office at 3-month follow-up, where a repeat duplex ultrasound revealed normal waveforms and velocities throughout the left arm. He was also seen in the office recently, and he is doing well with no new complaints.

## Discussion

Most subclavian artery dissections, pseudoaneurysms, and AVFs are most commonly seen after traumas [4]. Incidences of traumatic AVFs, dissections, and pseudoaneurysms of 6.8% up to 21.2% in patients with arterial lesions have been reported in the literature with most located in the lower extremities near long bones [3]. Subclavian artery injuries are rare with an incidence of 1%–2% [5]. Digital subtraction arteriography is the gold standard for diagnosing AVF although less invasive methods such as duplex or CT imaging allow quick and efficient diagnosis.

In terms of anatomy, the medial portion of the clavicle has been measured to be as close as 4.8 mm to the subclavian vein and between 11 and 22 mm from the subclavian artery [6]. This close proximity heightens the risk of inadvertent trauma to these vessels intraoperatively causing complications such as dissections, ruptures, pseudoaneurysms, or AVFs. The time interval between injury and presentation may vary from days to years and some patients remain asymptomatic [3]. Injuries in this location can especially be fatal because the clavicle hinders proper compression to achieve hemostasis. It is suspected that the injury in our patient may have developed secondary to trauma of the left clavicle during the initial surgical resection. The subclavian artery dissection likely developed into a pseudoaneurysm, which subsequently fistulized to the subclavian vein resulting in the AVF. Immediate complications of AVFs may include arterial and venous dilation proximal to the fistula, fistula rupture, and thrombosis. Longstanding AVFs can progress to high output congestive heart failure, murmurs, and thrills on physical examination [7]. Timely intervention is recommended to prevent these complications as the shunt volume gradually increases with time.

AVFs have historically been treated with open surgical repair that can be associated with significant morbidity and mortality especially in patients with comorbid medical conditions [8, 9]. In this patient, his comorbid medical conditions put him at a higher risk of complications from major surgical procedures such as open surgical repair. Technological advances have introduced less invasive techniques for treating traumatic pseudoaneurysms such as external ultrasound compression, transcatheter coil embolization, endovascular repair, and ultrasound-guided thrombin injections [8, 10, 11]. The use of endovascular balloon angioplasty and stent–graft placement in our patient was able to reduce the amount of blood loss, damage to surrounding structures, and length of hospital stay. The use of endovascular stent–grafts allows the surgeon to circumvent difficult anatomy by eliminating the need for open surgery [12–17].

There is a paucity of research on the incidence and management of Subclavian AVF. To our knowledge, traumatic fistula between the left subclavian artery and the subclavian vein has not been adequately reported in current literature. A high index of suspicion of subclavian AVFs should remain, especially in the setting of a traumatic event with subsequent development of a pseudoaneurysm. This case highlights the importance of recognizing this complication early to prevent long-term complications.

## Conclusion

Subclavian AVF is rare and may develop after surgical procedures or trauma. Suspicion for subclavian AVF should remain high and endovascular repair with a covered stent is a safe and effective surgical treatment method over open surgical repair.

## Limitations

Limitations of our case report include our short-term follow-up. A longer-term follow-up would be beneficial in elucidating the sequelae of the traumatic AVF of the subclavian artery and vein post-endovascular balloon and stenting. Further documented cases of rare presentations of traumatic subclavian AVFs may help provide further understanding of the nature of the disease and bring awareness to the vascular and trauma surgery community.
